# Supplementary material for: Succinate mediates inflammation-induced adrenocortical dysfunction
Source: eLife. 2023 Jul 14;12:e83064. doi: 10.7554/eLife.83064 (PMC10374281; doi:10.7554/eLife.83064)
Supplement: Figure 5—figure supplement 2—source data 1. [file elife-83064-fig5-figsupp2-data1.zip › Figure5-Suppl2_SourceData1.pptx]

## Slide 1
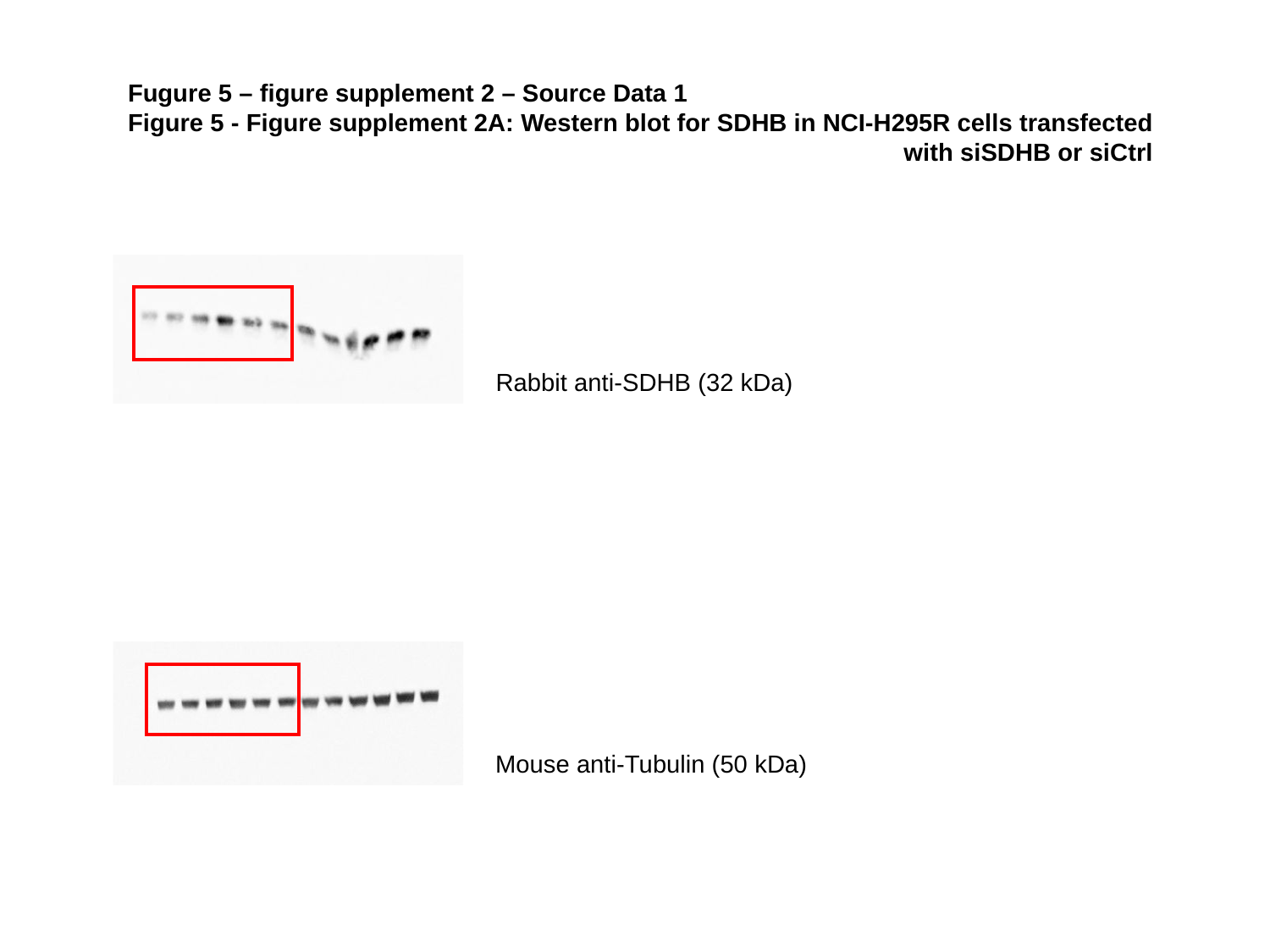

Fugure 5 – figure supplement 2 – Source Data 1
Figure 5 - Figure supplement 2A: Western blot for SDHB in NCI-H295R cells transfected
						 with siSDHB or siCtrl
Rabbit anti-SDHB (32 kDa)
Mouse anti-Tubulin (50 kDa)

## Slide 2
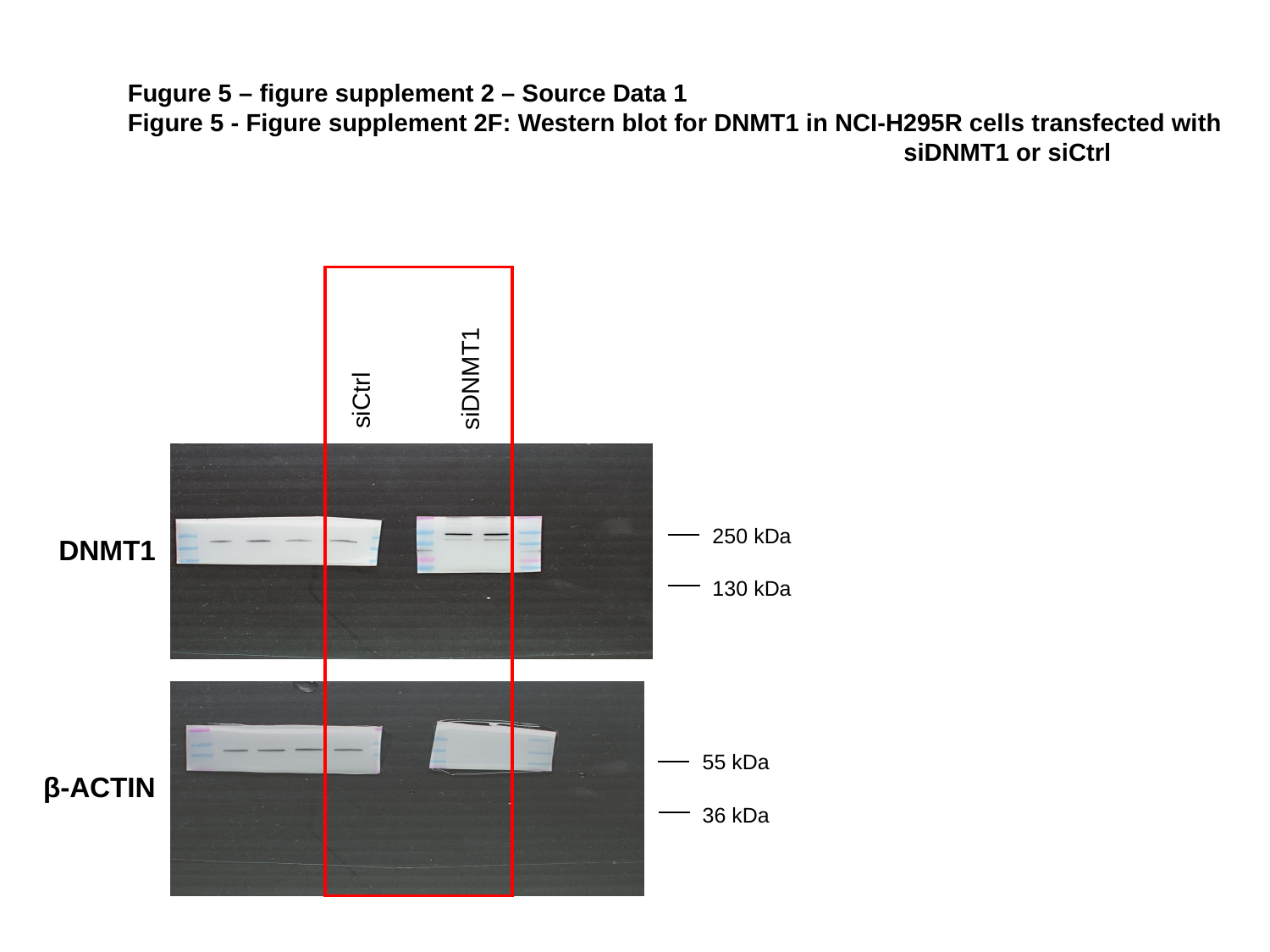

Fugure 5 – figure supplement 2 – Source Data 1
Figure 5 - Figure supplement 2F: Western blot for DNMT1 in NCI-H295R cells transfected with
						 siDNMT1 or siCtrl
siDNMT1
siCtrl
250 kDa
DNMT1
130 kDa
55 kDa
β-ACTIN
36 kDa
