# Supplementary material for: Succinate mediates inflammation-induced adrenocortical dysfunction
Source: eLife. 2023 Jul 14;12:e83064. doi: 10.7554/eLife.83064 (PMC10374281; doi:10.7554/eLife.83064)
Supplement: Figure 6—source data 1. [file elife-83064-fig6-data1.zip › Figure6_SourceData5.pptx]

## Slide 1
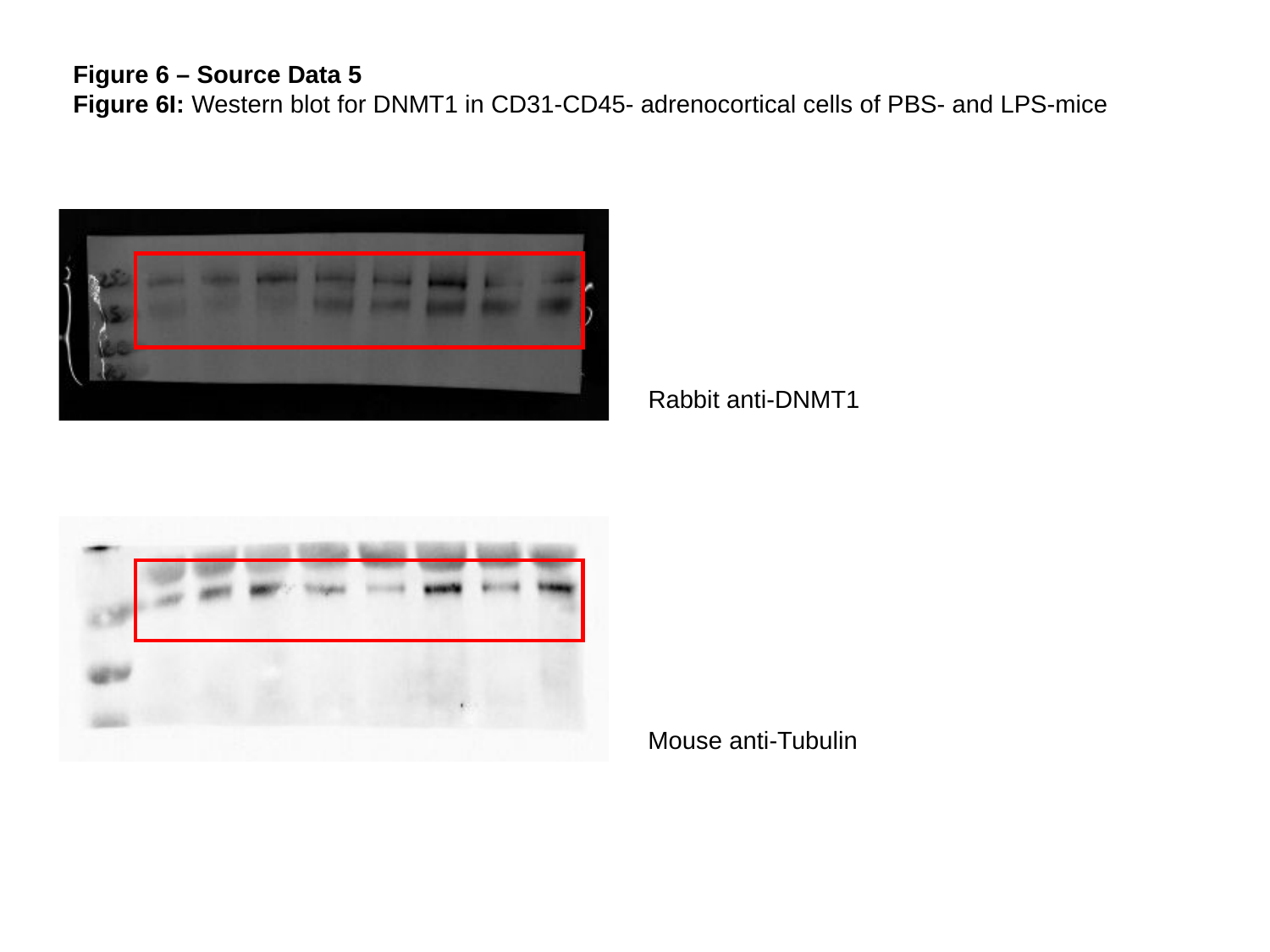

Figure 6 – Source Data 5
Figure 6I: Western blot for DNMT1 in CD31-CD45- adrenocortical cells of PBS- and LPS-mice
Rabbit anti-DNMT1
Mouse anti-Tubulin
