# Supplementary material for: Succinate mediates inflammation-induced adrenocortical dysfunction
Source: eLife. 2023 Jul 14;12:e83064. doi: 10.7554/eLife.83064 (PMC10374281; doi:10.7554/eLife.83064)
Supplement: Figure 6—source data 1. [file elife-83064-fig6-data1.zip › Figure6_SourceData6.pptx]

## Slide 1
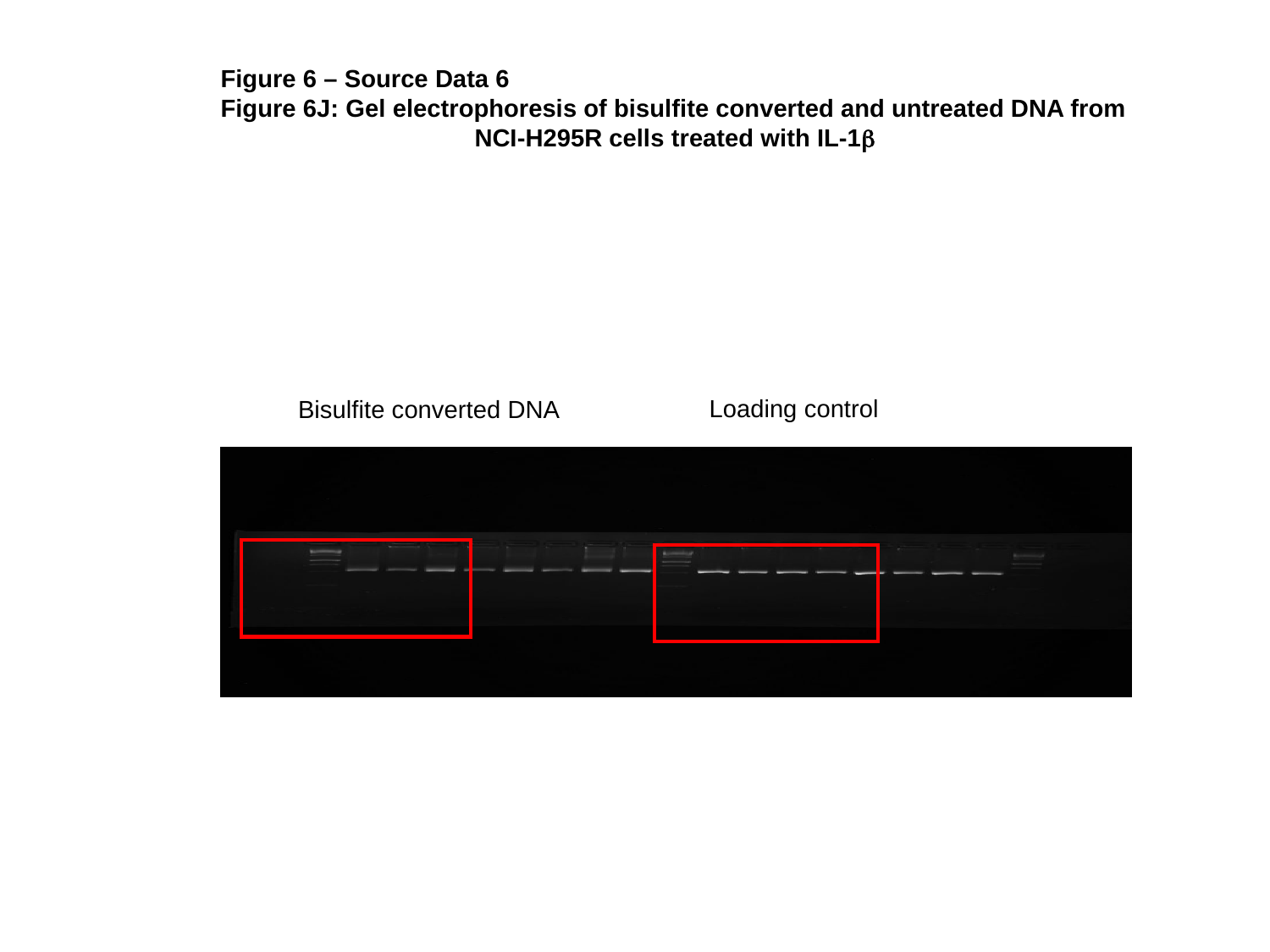

Figure 6 – Source Data 6
Figure 6J: Gel electrophoresis of bisulfite converted and untreated DNA from
		NCI-H295R cells treated with IL-1b
Loading control
Bisulfite converted DNA
